# Supplementary material for: Learning curve and surgical outcome of robotic assisted colorectal surgery with ERAS program
Source: Sci Rep. 2022 Nov 29;12:20566. doi: 10.1038/s41598-022-24665-w (PMC9709162; doi:10.1038/s41598-022-24665-w)
Supplement: Supplementary file 1 — Supplementary Information 1. [file 41598_2022_24665_MOESM1_ESM.zip › LSC Raw data-Ñ[▒K/053/OP N_ñw╝╨░O▒Kñσ.pdf]

W55 / W55

臺中榮民總醫院

手術室護理記錄

[redacted]

基本資料

- 室別： 開刀房-05室，手術科別： GS+CRS，手術類別： 常規，日期： 2019/11/05
- 進入時間： 1710 ； 麻醉開始時間： 1745 ； Time Out時間： 1825 ； 劃刀時間： 1826 ； 手術結束時間： 0020 送出時間： 0030 ；
- 麻醉方式： GE

  診斷：gallstones

  手術名稱：Laparoscopic Cholecystectomy

  手術局部用藥：NIL

  術前預防性抗生素：
  - 抗生素名稱一： Veterin 1GM IVD由麻醉護理師執行
  - 時間： 1815
  - 上次簽章: 黃珮涵

  ，追加預防性抗生素：
  - 抗生素名稱一： CEFA 1GM IVD由麻醉護理師執行
  - 時間： 2215
  - 上次簽章: 黃珮涵
- 手術醫師： 吳峰旭，林俊余，林昌霖，江世偉，林陳彥
- 麻醉醫師： VS 沈靜慧
-
  - 刷手護士A ： 林怡廷，時間： 1710，接替刷手一： 邱曉玲，時間： 1800，接替刷手二： 顧欣宜，時間： 2130
  - 流動護士A ： 邱曉玲，時間： 1710，接替流動一： 詹佳穎，時間： 1730，接替流動二： 黃珮涵，時間： 1800
- 更多接替及流動護士： 接替刷手三： 邱儷嫙，時間： 2200，接替刷手四： 阮鈺珊，時間： 2300，接替流動三： 呂昭蓉，時間： 2300

皮膚狀況

A-V Shunt： 無

術前： 完整

記錄者： 上次簽章: 詹佳穎

術後： 完整

記錄者： 上次簽章: 呂昭蓉

手術準備

手術臥式 單一手術臥式

-
  - 手術臥式： 仰臥
  - 使用附件： 溫毯 38 ℃，頭圈
  - 皮膚準備： 10%Aq Betadine，Hibiscrub，其它： 2%chlorhexidine
  - 電燒： 使用電燒: 電極片位置： 左大腿，記錄者： 上次簽章: 呂昭蓉

止血帶T： 無

植入物

- 植入物名稱/號碼/數量/部位：Endo GIA 60*4.1mm*3/腸吻合處

，記錄者： 上次簽章: 呂昭蓉

標本送檢

- 細菌培養 無
- 細胞學 無
- 冰凍切片 無
- 病理標本

組織名稱一： GB
,
組織名稱二： A-D colon

- 截肢 無

標本交付 標本： 2 袋 , 標本： 0 盒 , 標本： 0 瓶 , 細胞學： 0 管 , Culture Tube： 0 管 , 被交付醫師： CR 江世偉 ,

- 記錄者： 上次簽章: 呂昭蓉
- 時間： 0030

傷口術後敷料

傷口敷料： 傷口敷料： 無

- 請於圖上標示部位：

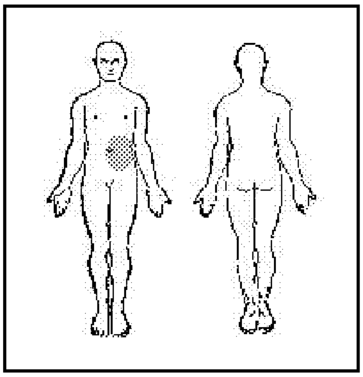

- 傷口敷料： 紗布

導管

導管	天數	導管資訊	插入日期	插入時間	更換日期	拔除日期	拔除時間	拔除原因	預計拔除/更換	預計拔除/更換日期
Foley(1)	2	號碼:16 Fr.; 部位:膀胱;	2019/11/05	1800						
Penrose Drain(1)	2	號碼:12 mm.; 部位:脾窩; 註記:左腹;	2019/11/05	1145						
Penrose Drain(1)	2	號碼:12 mm.; 部位:肝下; 註記:右腹;	2019/11/05	1140						
Sump NG Tube(1)	2	號碼:16 Fr.; 部位:胃;	2019/11/05	1815						

用物計數

- 手術中出血量： 450 c.c.
- 手術中尿量： 350 c.c.
-
    - 其它： 無

用物計數： 多切口同時縫合

- 1.器械： 正確 , 2.縫針： 正確 , 3.刀片： 正確 , 4.紗布： 正確 , 5.腹部墊： 未使用 , 6.紗球： 未使用 , 7.PEANUTS ： 未使用 , 8.紗條： 未使用 , 9.絲帶： 未使用 , 10.針頭： 未使用 , 11.Vessel loop： 未使用 , 12.Boots： 未使用 , 13.Jelco： 未使用 , 14.棉條： 未使用 ,
    - 第一次：
        - 刷手護士 ： 阮鈺珊
        - 流動護士： 上次簽章: 呂昭蓉
        - 時間： 0005

    ,
    - 第二次：
        - 刷手護士 ： 阮鈺珊
        - 流動護士： 上次簽章: 呂昭蓉
        - 時間： 0010

    ,
    - 第三次：
        - 刷手護士 ： 阮鈺珊

- 流動護士： 上次簽章: 呂昭蓉
- 時間： 0015

備註

備註：NIL

- 記錄者： 上次簽章: 呂昭蓉
- 時間： 0030

填寫護理人員 呂昭蓉

正式摘要

W55 / W55

doctorRank:B
